# Supplementary material for: Association between the rs1544410 polymorphism in the vitamin D receptor (VDR) gene and insulin secretion after gestational diabetes mellitus
Source: PLoS One. 2020 May 14;15(5):e0232297. doi: 10.1371/journal.pone.0232297 (PMC7224565; doi:10.1371/journal.pone.0232297)
Supplement: Attachment — Submitted filename: Response to Reviewers.doc [file pone.0232297.s002.doc]

**Response to Reviewers**

**Academic Editor:**

I request the authors to clear the following queries

**1.** Explain how the study size was arrived at.

**Authors’ reply:** Briefly, women delivering between 2003–2005 were invited to participate in the study, covering 86% of all pregnancies in the County of Skane in southern Sweden, including four of ﬁve delivery departments. The diagnosis of GDM was made using a 75-g oral glucose tolerance test (OGTT) at the twenty-eighth and/or the twelfth week of gestation. In the original evaluation, GDM was deﬁned as two-hour capillary plasma glucose ≥10.0 mmol/l, gestational IGT as two-hour capillary plasma glucose 8.6–9.9 mmol/l, and normal glucose tolerance (NGT) during pregnancy as two-hour capillary plasma glucose <8.6 mmol/l. Among those who accepted to participate in the study and after exclusion of those who had already been diagnosed as having diabetes, 160 women had GDM, 309 had Gestational IGT and 167 had NGT. Women were followed for the development of diabetes using an OGTT at 1–2 years and 5 years after pregnancy, or until the diagnosis of diabetes. The World Health Organization (WHO) 1999 diagnostic criteria were used for classification of diabetes during follow-up. Measurements of both glucose and insulin concentrations at 0, 30, and 120 min during OGTT at 1‒2 years postpartum were performed to calculate indices of beta-cell function and insulin resistance, as previously reported. Serum samples for determination of 25(OH)D3 were collected during the OGTT at 1‒2 years postpartum. In the present investigation, we used the diagnostic criteria for GDM recommended by the WHO in 1999. Based on these criteria and on successful measurements of 25OHD3 concentrations, we identified 376 women who had previously had GDM and who formed the basis of the present study.

We have now revised the Section “Material and methods/Patients” accordingly.

**2.** Did the authors confirmed the genotyping results using any other methods (sequencing etc.)

**Authors’ reply:** As the *TaqMan Allelic Discrimination Assay* is well validated method for SNP genotyping, we have not used any other methods to confirm the genotyping. However, we used positive controls during the genotyping. Moreover, the genotyping results were confirmed by re-genotyping of about 20% of the samples using the same genotyping method.

We think this would be enough to confirm our results and hope it is accepted according to your journal policies. We have now added this information to the Section “Material and methods/Genetic analysis”.

**3.** Please include the genotyping raw data (spss file etc.) as supplementary information.

**Authors’ reply:** We would like to bring to your attention that we discovered that two samples of the studied samples were not genotyped because of no available DNA. We have now added this information to the Section “Material and methods/Genetic analysis”. The genotyping raw data is included in SPSS file as supplementary information. Please, let us to know if you need more raw data (t. ex. original genotyping raw data from the *TaqMan ® Genotyper ™ Software*).

- If you would like to make changes to your financial disclosure, please include your updated statement in your cover letter.

**Authors’ reply:** No changes to our financial disclosure have been made.

- To enhance the reproducibility of your results, we recommend that if applicable you deposit your laboratory protocols in protocols.io, where a protocol can be assigned its own identifier (DOI) such that it can be cited independently in the future. For instructions see: [http://journals.plos.org/plosone/s/submission-guidelines#loc-laboratory-protocols](http://journals.plos.org/plosone/s/submission-guidelines#loc-laboratory-protocols)

**Authors’ reply:** The genotyping protocol have been deposited in protocols.io. The DOI is: dx.doi.org/10.17504/protocols.io.bcjbiuin

Please include the following items when submitting your revised manuscript:

• A rebuttal letter that responds to each point raised by the academic editor and reviewer(s). This letter should be uploaded as separate file and labeled 'Response to Reviewers'.

**Authors’ reply:** This is done.

• A marked-up copy of your manuscript that highlights changes made to the original version. This file should be uploaded as separate file and labeled 'Revised Manuscript with Track Changes'.

**Authors’ reply:** This is done.

• An unmarked version of your revised paper without tracked changes. This file should be uploaded as separate file and labeled 'Manuscript'.

**Authors’ reply:** This is done.

**Journal Requirements:**

**1.** When submitting your revision, we need you to address these additional requirements.

Please ensure that your manuscript meets PLOS ONE's style requirements, including those for file naming. The PLOS ONE style templates can be found at

http://www.journals.plos.org/plosone/s/file?id=wjVg/PLOSOne_formatting_sample_main_body.pdf and <http://www.journals.plos.org/plosone/s/file?id=ba62/PLOSOne_formatting_sample_title_authors_affiliations.pdf>

**Authors’ reply:** Your journal requirements have been taken into consideration during revision of the manuscript.

**2.** We note that you are reporting an analysis of a microarray, next-generation sequencing, or deep sequencing data set. PLOS requires that authors comply with field-specific standards for preparation, recording, and deposition of data in repositories appropriate to their field. Please upload these data to a stable, public repository (such as ArrayExpress, Gene Expression Omnibus (GEO), DNA Data Bank of Japan (DDBJ), NCBI GenBank, NCBI Sequence Read Archive, or EMBL Nucleotide Sequence Database (ENA)). In your revised cover letter, please provide the relevant accession numbers that may be used to access these data. For a full list of recommended repositories, see http://journals.plos.org/plosone/s/data-availability#loc-omics or [http://journals.plos.org/plosone/s/data-availability#loc-sequencing](http://journals.plos.org/plosone/s/data-availability#loc-sequencing).

**Authors’ reply:** The data are uploaded. The EGA accession numbers are:

RNAseq: EGAS00001004042

GWAS: EGAS00001004044

Phenotype: EGAS00001004056

This information has been added to the revised manuscript.

**3.** Please note that PLOS does not permit references to “data/results not shown.” Authors should provide the relevant data within the manuscript, the Supporting Information files, or in a public repository. If the data are not a core part of the research study being presented, we ask that authors remove any references to these data.

**Authors’ reply:** We have now provided data within the manuscript. Please, see “Table 2” in the Results Section.

**4.** Thank you for stating the following in the Acknowledgments Section of your manuscript:

"The study was supported by grants from the Research Funds of Skåne University Hospital and

289 the Skåne County Council Research and Development Foundation. The funders had no role in

290 study design, data collection and analysis, decision to publish, or preparation of the manuscript.".

**Authors’ reply:** We have got a little bit confused. You request in the next point that funding information should not appear in the Acknowledgments section or other areas of our manuscript. Please let us know if we have misunderstood your requirements. Please, see our response to the next point.

- We note that you have provided funding information that is not currently declared in your Funding Statement. However, funding information should not appear in the Acknowledgments section or other areas of your manuscript. We will only publish funding information present in the Funding Statement section of the online submission form.

* Please remove any funding-related text from the manuscript and let us know how you would like to update your Funding Statement. Currently, your Funding Statement reads as follows:

"The funders had no role in study design, data collection and analysis, decision to publish, or preparation of the manuscript.".

**Authors’ reply:** We have removed any funding-related text from the manuscript. We want to update our funding information as follow: “The study was supported by grants from the Research Funds of Skåne University Hospital, the Skåne County Council Research and Development Foundation and ALF Region Skåne. The funders had no role in study design, data collection and analysis, decision to publish, or preparation of the manuscript.”

**Reviewers' comments:**

Reviewer's Responses to Questions

Comments to the Author

**1.** Is the manuscript technically sound, and do the data support the conclusions?

The manuscript must describe a technically sound piece of scientific research with data that supports the conclusions. Experiments must have been conducted rigorously, with appropriate controls, replication, and sample sizes. The conclusions must be drawn appropriately based on the data presented.

Reviewer #1: Yes

Reviewer #2: Yes

**Authors’ reply:** There are no questions raised by the reviewer.

________________________________________

**2.** Has the statistical analysis been performed appropriately and rigorously?

Reviewer #1: Yes

Reviewer #2: Yes

**Authors’ reply:** There are no questions raised by the reviewer.

________________________________________

**3.** Have the authors made all data underlying the findings in their manuscript fully available?

The PLOS Data policy requires authors to make all data underlying the findings described in their manuscript fully available without restriction, with rare exception (please refer to the Data Availability Statement in the manuscript PDF file). The data should be provided as part of the manuscript or its supporting information, or deposited to a public repository. For example, in addition to summary statistics, the data points behind means, medians and variance measures should be available. If there are restrictions on publicly sharing data—e.g. participant privacy or use of data from a third party—those must be specified.

Reviewer #1: Yes

Reviewer #2: Yes

**Authors’ reply:** There are no questions raised by the reviewer.

________________________________________

**4.** Is the manuscript presented in an intelligible fashion and written in standard English?

PLOS ONE does not copyedit accepted manuscripts, so the language in submitted articles must be clear, correct, and unambiguous. Any typographical or grammatical errors should be corrected at revision, so please note any specific errors here.

Reviewer #1: Yes

Reviewer #2: Yes

**Authors’ reply:** There are no questions raised by the reviewer.

________________________________________

**5.** Review Comments to the Author

Please use the space provided to explain your answers to the questions above. You may also include additional comments for the author, including concerns about dual publication, research ethics, or publication ethics. (Please upload your review as an attachment if it exceeds 20,000 characters)

**Reviewer #1:** The study aimed to investigate possible associations of polymorphisms in genes involved in vitamin D metabolism with indices of insulin resistance and insulin secretion, and also with development of diabetes after gestational diabetes mellitus (GDM), the manuscript was found interesting, The subject selection and how subjects move through the study is clear. It is a good work.

**Authors’ reply:** There are no questions raised by the reviewer.

**Reviewer #2:** I would like to appreciate the work done by the authors submitted for publication. Overall the manuscript is well designed, experimental study is well executed, statistical data analysis performed is acceptable. Overall the manuscript is well written and the subject of study and findings reported are relevant.

**Authors’ reply:** There are no questions raised by the reviewer.

________________________________________

**6.** PLOS authors have the option to publish the peer review history of their article (what does this mean?). If published, this will include your full peer review and any attached files.

If you choose “no”, your identity will remain anonymous but your review may still be made public.

Do you want your identity to be public for this peer review? For information about this choice, including consent withdrawal, please see our Privacy Policy.

Reviewer #1: Yes: Moushira Erfan Zaki

Reviewer #2: No

[NOTE: If reviewer comments were submitted as an attachment file, they will be attached to this email and accessible via the submission site. Please log into your account, locate the manuscript record, and check for the action link "View Attachments". If this link does not appear, there are no attachment files to be viewed.]

While revising your submission, please upload your figure files to the Preflight Analysis and Conversion Engine (PACE) digital diagnostic tool, https://pacev2.apexcovantage.com/. PACE helps ensure that figures meet PLOS requirements. To use PACE, you must first register as a user. Registration is free. Then, login and navigate to the UPLOAD tab, where you will find detailed instructions on how to use the tool. If you encounter any issues or have any questions when using PACE, please email us at figures@plos.org. Please note that Supporting Information files do not need this step.

________________________________________

In compliance with data protection regulations, you may request that we remove your personal registration details at any time. (Remove my information/details). Please contact the publication office if you have any questions.
